# Artificial intelligence as a tool for improving health literacy in kidney care

**DOI:** 10.1371/journal.pdig.0000746

**Published:** 2025-02-21

**Authors:** Jing Miao, Charat Thongprayoon, Kianoush B. Kashani, Wisit Cheungpasitporn

**Affiliations:** 1 Division of Nephrology and Hypertension, Department of Medicine, Mayo Clinic, Rochester, Minnesota, United States of America; 2 Division of Pulmonary and Critical Care Medicine, Department of Medicine, Mayo Clinic, Rochester, Minnesota, United States of America; National Tsing-Hua University: National Tsing Hua University, TAIWAN

## Introduction

Understanding medical information is essential for patients to make informed healthcare decisions, and it directly influences health outcomes [1]. However, a significant portion of the population, including in the United States, faces challenges with health literacy. The National Assessment of Adult Literacy Survey has revealed that only 12% of American adults possess proficient health literacy skills necessary for comprehending the intricate of healthcare, while approximately 36% have basic or below levels of health literacy [2]. The typical American reads at an eighth-grade level [3], posing a challenge for medical professionals to convey information that accommodates various literacy levels.

To bridge this literacy gap, tailored communication strategies are essential to ensure all patients, regardless of their literacy level, understand the nuances of their health. Emerging technologies, such as generative artificial intelligence (AI) models like ChatGPT, are proving invaluable in this regard. These AI tools can simplify medical information into more digestible language, enhancing patient comprehension across several medical domains [4,5].

Recognizing the role of responsible AI in kidney health, the American Society of Nephrology (ASN) has launched the Partnership for Responsible Augmented Intelligence in Kidney Health in 2024 [6]. This initiative aims to cultivate a community of stakeholders dedicated to the ethical use of AI to improve health literacy and patient care in key areas such as acute kidney injury, chronic kidney disease, dialysis, and transplantation.

While the integration of AI in healthcare presents new avenues for enhancing patient outcomes in nephrology, it also brings challenges that need careful consideration. These include issues of equity, implementation, and effectively engaging patients. In this article, we focus not only on employing AI to close the literacy gap in kidney care, but also on navigating the ethical dimensions to ensure that advancements in AI lead to equitable improvements in patient education and health outcomes.

## Simplifying complex medical terminology

In our practice, patients dealing with kidney diseases, such as glomerular diseases, frequently struggle with complex medical terminology. Diagnoses like “focal segmental glomerulosclerosis” and “membranous nephropathy” exemplify terms that can overwhelm patients. These conditions involve intricate kidney pathophysiology and functions that are challenging to simplify for individuals without a medical background.

AI holds promise in alleviating these communication hurdles by converting such intricate medical terms into more understandable language. For instance, AI could clarify that “focal segmental glomerulosclerosis” involves scarring in sections of the kidney’s filtering units, potentially impacting kidney function. Likewise, it might explain “membranous nephropathy” as a thickening of the kidney’s filters due to immune responses, which could affect kidney performance. By providing these explanations in a clearer manner, AI can assist patients in comprehending their conditions better, thereby empowering them to make more informed decisions regarding their healthcare.

## Recent AI developments in enhancing health literacy in kidney care

In our recent work, we reported the utility of AI in augmenting health literacy within the context of kidney care. One study particularly focused on the role of an advanced AI language model, ChatGPT, in simplifying the medical jargon associated with living kidney donation [7]. Our findings indicate that the AI was able to reduce the reading complexity of medical documents from a ninth-grade level to approximately seventh-grade and fourth-grade levels using GPT-3.5 and GPT-4 models, respectively. This adjustment in readability is pivotal for extending the reach of medical information to those with varying levels of education, thereby fostering greater inclusivity in healthcare settings.

Furthering this effort, another study concentrated on utilizing AI to enhance health equity, specifically targeting the Hispanic communities through kidney transplantation education [8]. We employed ChatGPT to translate essential information regarding kidney transplantation from English to Spanish. This adaptation addressed significant barriers faced by Hispanic communities in accessing medically accurate and culturally attuned information. The translations achieved by the models were not only linguistically precise, but also culturally appropriate, as evidenced by a Cohen’s Kappa inter-rater reliability score of 0.85 assessed by native Spanish-speaking nephrologists.

These initiatives highlight AI’s potential to bridge health literacy gaps, especially among underserved groups, ensuring equitable health outcomes and informed choices for all, regardless of language or education.

## Ethical considerations in AI-Driven health literacy enhancements

As we integrate AI into our nephrology practice to enhance health literacy, it is imperative to consider the ethical implications [9,10]. These connotations include (1) *Equity and Access:* AI has the potential to identify and address disparities in kidney care, enhancing treatment delivery. Nevertheless, it is vital to craft AI tools that minimize, not magnify, existing healthcare gaps. Ethical guidelines for AI should focus on fairness and transparency to avoid increasing inequalities. (2) *Informed Consent and Autonomy:* Patients need clear information on how AI tools are used to educate them about their health conditions and treatment choices, ensuring it supports rather than undermines their autonomy. It is critical that AI complements patient input in decision-making, maintaining their central role. (3) *Bias and Fairness in AI Algorithms:* There is a risk of bias in AI algorithms, potentially leading to uneven treatment outcomes, particularly for minority groups. Rigorous testing and ethical standards must be applied to AI applications to correct biases and ensure equitable benefits for all patients. Finally, (4) *impact on Patient-Provider Relationships:* While AI can tailor health information effectively, it may lessen the personal interaction between patients and providers. It is essential to balance AI use with maintaining a personal, empathetic approach to care, preserving the crucial human element in healthcare.

## Conclusion

The incorporation of AI into nephrology represents a significant advancement in enhancing health literacy. By simplifying medical jargon and augmenting the accessibility of information, AI tools have the potential to close the health literacy gap, thereby empowering patients to make more informed decisions about their healthcare. Nevertheless, the effectiveness of these AI-driven initiatives is contingent upon tackling ethical issues and ensuring that the deployment of such technologies fosters equity and improves patient care. The ASN’s commitment to responsible AI utilization underscores the growing recognition within the nephrology community of the need for careful and considerate implementation of AI. This approach is vital for securing optimal health outcomes for all patients, marking a pivotal step in the evolution of kidney healthcare practices.
